# Global trends and prospects about inflammasomes in stroke: a bibliometric analysis

**DOI:** 10.1186/s13020-021-00464-9

**Published:** 2021-07-07

**Authors:** Junjun Yin, Jiayang Wan, Jiaqi Zhu, Guoying Zhou, Yuming Pan, Huifen Zhou

**Affiliations:** 1 Zhejiang Chinese Medical University, 548 Binwen Road, Hangzhou, 310053 Zhejiang China; 2 Peking University First Hospital, Beijing, 100034 China

**Keywords:** Ischemic stroke, Hemorrhagic stroke, Inflammasomes, Bibliometric, Mitochondrial dysfunction, Global trends

## Abstract

**Background:**

Sterile inflammation is a key pathological process in stroke. Inflammasome activation has been implicated in various inflammatory diseases, including ischemic stroke and hemorrhagic stroke. Hence, targeting inflammasomes is a promising approach for the treatment of stroke.

**Methods:**

We applied bibliometric methods and techniques. The Web of Science Core Collection was searched for studies indexed from database inception to November 26, 2020. We generated various visual maps to display publications, authors, sources, countries, and keywords.

**Results:**

Our literature search yielded 427 publications related to inflammasomes involved in stroke, most of which consisted of original research articles and reviews. In particular, we found that there was a substantial increase in the number of relevant publications in 2018. Furthermore, most of the publications with the highest citation rates were published in 2014. Relatively, the field about inflammasomes in stroke developed rapidly in 2014 and 2018. Many institutions contributed to these publications, including those from China, the United States, and worldwide. We found that NLR family pyrin domain containing 3 (NLRP3) was the most studied, followed by NLRP1, NLRP2, and NLRC4 among the inflammasomes associated with stroke. Analysis of keywords suggested that the most studied mechanisms involved dysregulation of extracellular pH, efflux of Ca^2+^ ions, dysfunction of K^+^/Na^+^ ATPases, mitochondrial dysfunction, and damage to mitochondrial DNA.

**Conclusions:**

Given the potential diagnostic and therapeutic implications, the specific mechanisms of inflammasomes contributing to stroke warrant further investigation. We used bibliometric methods to objectively present the global trend of inflammasomes in stroke, and to provide important information for relevant researchers.

## Introduction

Stroke is a major contributor to disability and death worldwide, and is characterized by a sudden decrease in blood flow to brain tissue that often leads to critical neurological impairment [11, 22]. Ischemic stroke (IS) and intracerebral hemorrhage (ICH) are major types of stroke. IS accounts for 80% of all cases of stroke [32]. Clinically, the treatment time window for patients with IS is only 3.0–4.5 h [4] Because of the narrow time window, brain injury caused by cerebral ischemia remains a serious problem [24]. Simultaneously, there is still no effective treatment for ICH [30]. Since the currently available methods for the treatment of brain injury after stroke are insufficient, an urgent identification and development of new treatment is needed.

In terms of mechanism, excessive inflammatory responses contributes to the progression of stroke and exacerbation of neurological impairment [27]. Inhibition of excessive inflammatory responses may represent a promising therapeutic strategy for the treatment of stroke. At present, inflammasomes, which are upstream inflammatory molecules, play an important role in cellular signaling underlying inflammatory responses [7]. Moreover, inflammasomes are important factors contributing to inflammatory diseases. Therefore, inhibiting abnormal activation of inflammasomes may represent a promising strategy for the treatment of stroke.

To date, nearly 10 inflammasomes have been described, including NLR family pyrin domin containing 1 (NLRP1), NLRP2, NLRP3, NLRP6, NLRC4, NLRP12, pyrin, absent in melanoma 2 (AIM2), and interferon gamma inducible protein 16 (IFI16). [2, 13, 15, 16, 18, 21] However, the specific inflammasomes that are most strongly associated with stroke and how they are activated have remained unclear, which promoted us to review and analyze the corresponding literature in the present study.

Bibliometrics is a widely accepted statistical method that is used to describe accumulated knowledge and trends in specific research areas [14]. In recent years, bibliometrics has been used to provide clear insights into many biomedical fields [26]. Moreover, these indicators can be used to help researchers understand global trends about inflammasomes in stroke. This review could lay the foundation of a new and interesting frontier.

## Methods

### Data collection and filtration

**Fig. 1:**
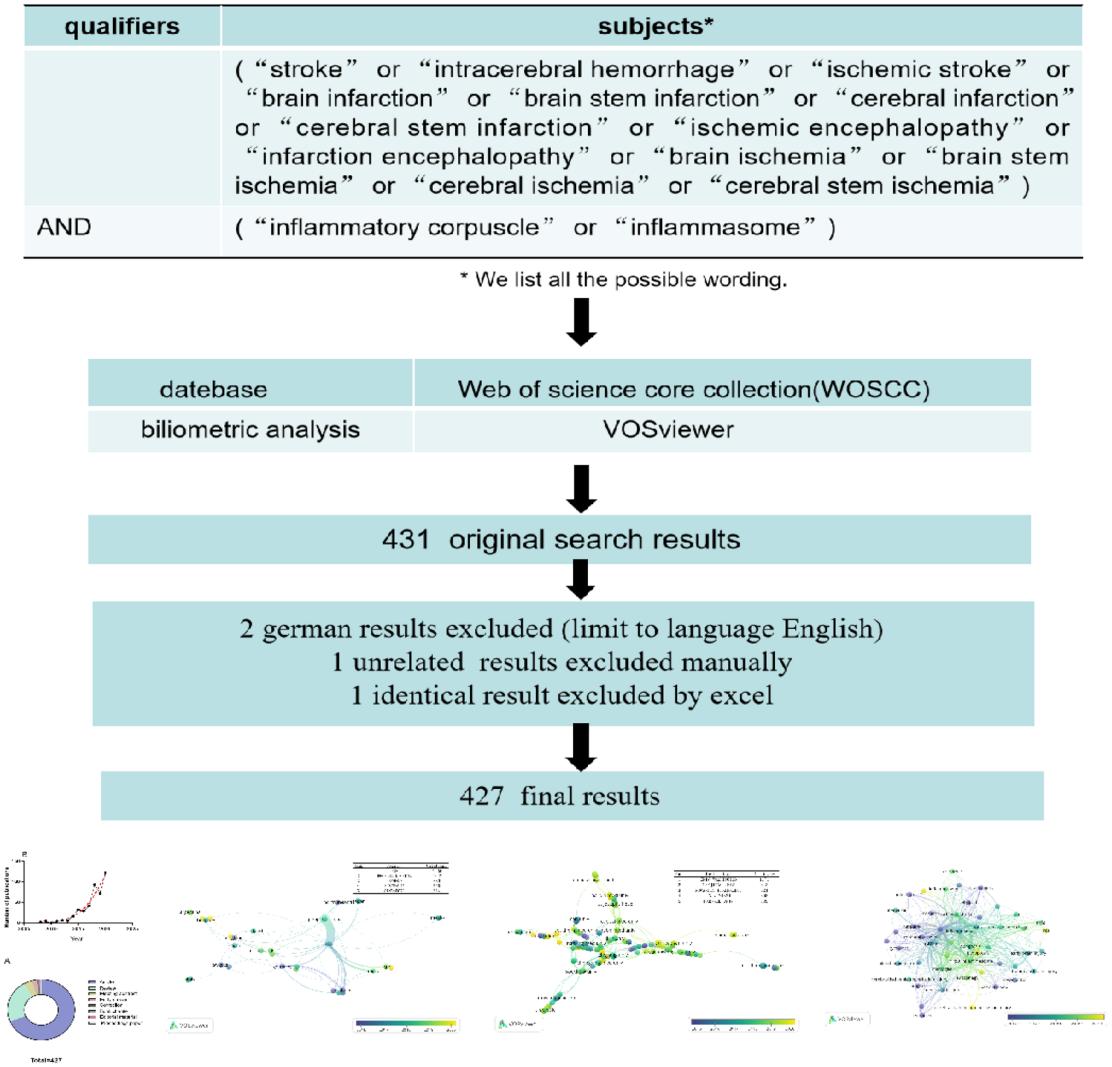
Details of filtrating the data. A manual review was performed on the original search on WOSCC to filter out documents that are not related to inflammasomes in stroke

We conducted a comprehensive literature search in the Web of Science Core Collection (WOSCC) from its inception (1985) to November 26, 2020. In order to yield as many results as possible, we use the following terms: “stroke” or “intracerebral hemorrhage” or “ischemic stroke” or “brain infarction” or “brain stem infarction” or “cerebral infarction” or “cerebral stem infarction” or “ischemic encephalopathy” or “infarction encephalopathy” or “brain ischemia” or “brain stem ischemia” or “cerebral ischemia” or “cerebral stem ischemia” AND “inflammatory corpuscle” or “inflammasome”. The language is restricted to English. There were no restrictions in terms of document type, data category, or document year. Following the above criteria, we obtained 431 original records, excluded four unrelated articles and finally got 427 results. The flowchart of the whole procedure was shown in Fig. 1.

### Data analysis

An Excel-based (Microsoft) analysis tool was used for data aggregation, and related figures were drawn with GraphPad Prism 8 software. VOSviewer, a visualization tool, enables researchers to create knowledge maps, assess the latest research advances, and identify research hotspots [31]. We used VOSviewer software (version 1.6.15) to analyze literature types, journal sources, authors, co-cited authors, countries, institutions, and key words to form a social network map. The relevant indicators are explained below. The citation attribute indicates the number of citations received by a document or the total number of citations received by all documents published by a source, author, organization, or country when working with co-authorship, citation, or bibliographic coupling links. The author co-cited attribute is defined as journals, authors, or references cited together by researchers. Finally, the occurrence attribute indicates the number of documents in which a keyword occurred when working with keywords (from VOSviewer Manual).

## Results

### Publication types and quantities

**Fig. 2:**
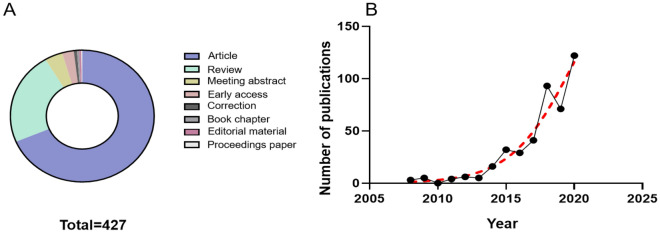
**A** The hollow pie chart illustrates the proportion of document number of each type. **B** The line chart shows the number of papers on inflammasome and stroke research published and the exponential trend line over time (the black line and red dotted line)

In the present study, a total of 427 publications related to the involvement of inflammasomes in stroke were identified. We collated and analyzed the statistical data included in each study and determined preliminary conclusions. Most of the publications were original research articles (302 publications), followed by review articles (103 publications) (Fig. 2A). Other publication types included conference abstracts (17 publications), early-access articles (14 publications), corrections (three publications), book chapters (two publications), editorial materials (two publications), and a proceedings publication (1 publication) (Fig. 2A). The large numbers of original research and review articles were then used to assess trends and insights into the association between inflammasomes and stroke.

The annual scientific research results of publications in this field—published from 2008 to November 26, 2020—are presented in Fig. 2B. The total number of articles increased from a minimum of 3 in 2008 to 122 in 2020 (through November 26, 2020). The number of publications per year showed continuous growth, as shown in Fig. 2B.

We found two turning points in this research field. First, in 2014, there were not many related publications (16 publications); however, the citation rates of the articles published in 2014 were relatively high (Table 1). We found two turning points in this research field. Second, there was a substantial increase in the number of related publications in 2018.

**Table 1:** The TOP 10 highly cited articles

Rank	Title	Total Citations	Authors	Source	Year	Type
1	Pyroptosis: host cell death and inflammation	1171	Bergsbaken T, Fink SL et al	NATURE REVIEWS MICROBIOLOGY Volume 7, issue 2, pages 99–109	2009	Review
2	Interleukin-1 beta inhibition and the prevention of recurrent cardiovascular events: Rationale and Design of the Canakinumab Anti-inflammatory Thrombosis Outcomes Study (CANTOS)	574	Ridker PM, Thuren T et al	AMERICAN HEART JOURNAL Volume162, issue 4, pages 597–605	2011	Article
3	Inflammasomes in the CNS	306	Walsh JG, Muruve DA et al	NATURE REVIEWS NEUROSCIENCE Volume 15, issue 2, pages 84–97	2014	Review
4	Intravenous immunoglobulin suppresses NLRP1 and NLRP3 inflammasome-mediated neuronal death in ischemic stroke	206	Fann DYW, Lee SY et al	CELL DEATH AND DISEASE Volume 4, e790	2013	Article
5	P2X7 receptor: an emerging target in central nervous system diseases	197	Sperlagh B, Illes P et al	TRENDS IN PHARMACOLOGICAL SCIENCES Volume 35, issue 10, pages 537–547	2014	Review
6	Inhibition of the inflammasome complex reduces the inflammatory response after thromboembolic stroke in mice	195	Abulafia DP,Vaccari, JPD et al	JOURNAL OF CEREBRAL BLOOD FLOW AND METABOLISM Volume 29, issue 3, pages 534–544	2009	Article
7	NLRP3 deficiency ameliorates neurovascular damage in experimental ischemic stroke	182	Yang F, Wang ZY et al	JOURNAL OF CEREBRAL BLOOD FLOW AND METABOLISM Volume 34, issue 4, pages 660–667	2014	Article
8	Release and activity of histone in diseases	173	Chen R, Kang R et al	CELL DEATH AND DISEASE Volume 5, e1370	2014	Review
9	Functions and mechanisms of microglia/macrophages in neuroinflammation and neurogenesis after stroke	165	Xiong XY, Liu L et al	PROGRESS IN NEUROBIOLOGY Volume 142, pages 23–44	2016	Review
10	Activation and regulation of cellular inflammasomes: gaps in our knowledge for central nervous system injury	153	Vaccari JPD, Dietrich WD et al	JOURNAL OF CEREBRAL BLOOD FLOW AND METABOLISM. Volume 34, issue 3, pages 369–375	2014	Review

### Highly cited articles

The 10-most highly cited articles, including 4 articles and 6 reviews, are shown in Table 1. All 10 of these articles were published between 2009 and 2016. Further analysis revealed that half of these articles were published in 2014. Therefore, we conclude that 2014 was a fast-growing year in published research on the role of inflammasomes in stroke.

### Journals

There were a total of 203 peer-reviewed journals that published manuscripts in this research field. The top-10 journals in terms of the total numbers of related publications are listed in Table 2. The most widely published journal was J NEUROINFLAMM (28 publications), followed by INT IMMUNOPHARMACOL (12 publications), INT J MOL SCI (12 publications), BIOCHEM BIOPH RES CO (10 publications), EXP NEUROL (10 publications), CNS NEUROSCI THER (9 publications), MOL NEUROBIOL (9 publications), J CEREBR BLOOD F MET (8 publications), STROKE (8 publications), and FRONT CELL NEUROSCI (7 publications). The number of papers published in each journal serve as a useful indicator of the interest of each journal in publishing articles related to the involvement of inflammasomes in stroke.

**Table 2:** The TOP 10 Journals of publications and co-cited journals

Rank	Journals	Publications	Impact factor	Co-cited journals	Citations	Impact factor
1	J NEUROINFLAMM	28	5.793	STROKE	846	7.190
2	INT IMMUNOPHARMACOL	12	3.943	J CEREBR BLOOD F MET	798	5.681
3	INT J MOL SCI	12	4.556	NATURE	680	42.778
4	BIOCHEM BIOPH RES CO	10	2.985	P NATL ACAD SCI USA	599	9.412
5	EXP NEUROL	10	4.691	J NEUROSCI	556	5.673
6	CNS NEUROSCI THER	9	4.074	J NEUROINFLAMM	532	5.793
7	MOL NEUROBIOL	9	4.500	J BIOL CHEM	515	4.238
8	J CEREBR BLOOD F MET	8	5.681	PLOS ONE	475	2.740
9	STROKE	8	7.190	J IMMUNOL	470	4.886
10	FRONT CELL NEUROSCI	7	3.921	CELL	392	38.637

Table 2 lists the top 10 journals in terms of their citations and impact factors in 2020 (Table 2). Co-citation journals were defined as journals jointly cited by researchers, and they represent the popularity of journals by researchers investigating the role of inflammasomes in stroke. STROKE topped the list with 846 citations. J CEREBR BLOOD F MET ranked second, with a total of 798 citations and an impact factor of 5.681. NATURE was cited 680 times, and its impact factor was 42.778. J NEUROINFLAMM, J CEREBR BLOOD F MET, and STROKE were also journals that were clearly of interest to this field. It is noteworthy that the number of publications in this field was relatively small, and even J NEUROINFLAMM (which published the most articles) only had 28 related publications. However, we suspect that the numbers of related articles published in these journals will increase significantly in the coming years due to increased interest in investigating the role of inflammasomes in stroke.

### Author

A total of 2009 authors participated in the inflammasomes in stroke study. We list the top 10 authors and co-cited authors according to the citations in Table 3. The top 3 authors in citations were as follows: (1) KEANE RW (479 citations), (2) VACCARI JPD (441 citations), (3) DIETRICH WD (428 citations). Similarly, the co-cited authors rank 1–10 were FANN DYW, MARTINON F, YANG F, LAMKANFI M, ZHOU RB, VACCARI JPD, ABULAFIA DP, SCHRODER K, DENES A and HENEKA MT.

**Table 3:** The TOP 10 authors and co-cited author

Rank	Authors	Citations^#^	H-index*	Co-cited authors	Citations
1	KEANE RW	479	38	FANN DYW	214
2	VACCARI JPD	441	25	MARTINON F	126
3	DIETRICH WD	412	91	YANG F	99
4	BROUGH D	367	48	LAMKANFI M	92
5	CHEN S	361	15	ZHOU RB	81
6	ZHANG JH	308	73	VACCARI JPD	74
7	ARUMUGAM TV	304	56	ABULAFIA DP	71
8	ALLAN SM	289	45	SCHRODER K	70
9	ISHRAT T	184	32	DENES A	68
10	ZHAO J	180	13	HENEKA MT	67

^#^Citations refer to the sum of citations that each author’s articles related to “inflammasomes in stroke” research have totally received

^*^H-Index, a scientific evaluation of influence, is extracted from WOSCC

### Countries

**Fig. 3:**
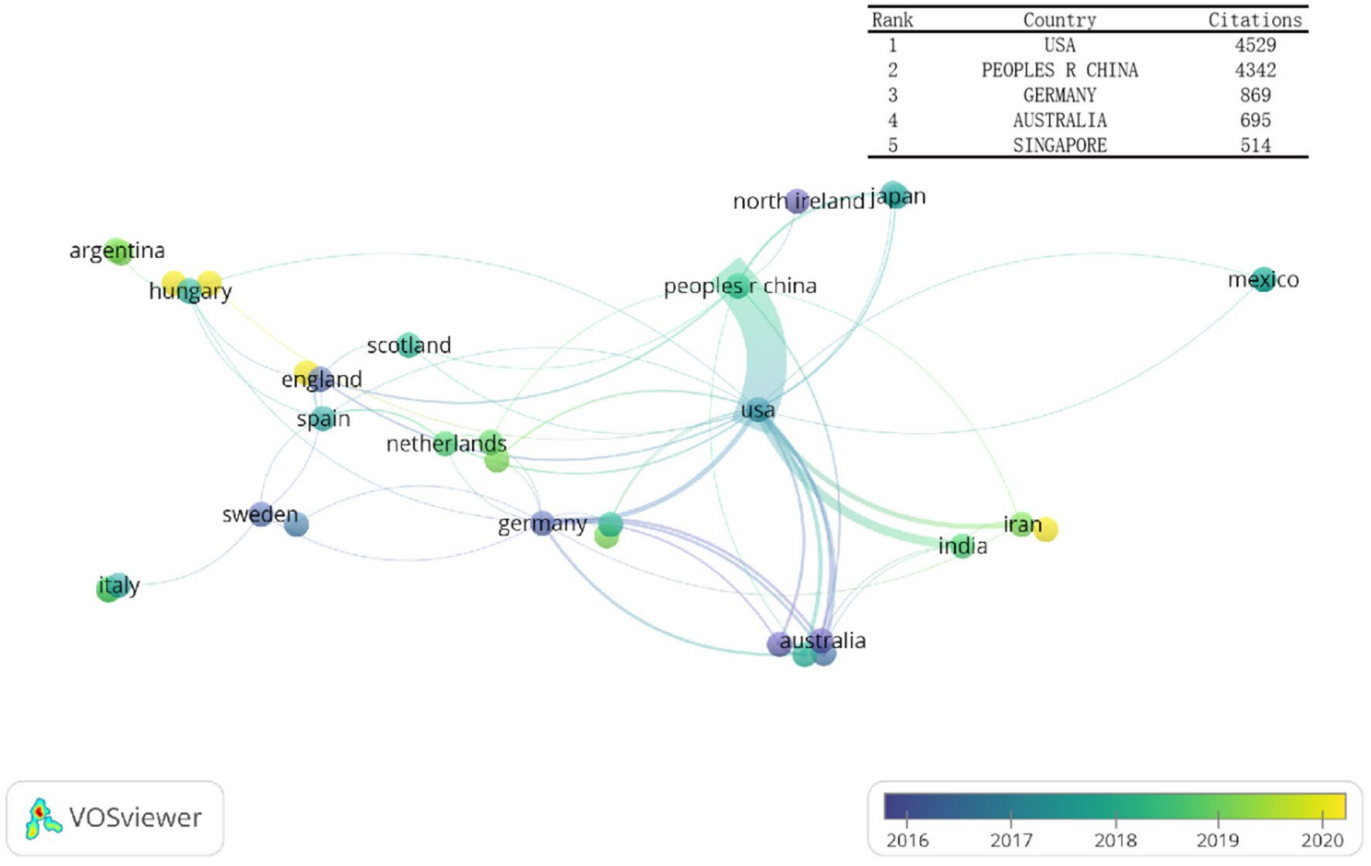
Co-authorship country analysis. The top 5 citations countries and the country co-authorship overlay visualization map. The color of each circle indicates the average publication year for the country, according to the color gradient shown in the lower right corner

We found that a total of 37 countries contributed to publications related to the role of inflammasomes in stroke. The top5 countries/regions were as follows: (1) United States (4529 citations); (2) China (4342 citations); (3) Germany (869 citations); (4) Australia (695 citations); and (5) Singapore (514 citations). In addition, we used VOSviewer to analyze the co-authorship of each country. In the figure, the color of the node represents the average year of publications; the thickness of each line represents the scale of cooperation between countries (Fig. 3). We found that China and the United States were the most active countries in this field, and that these two countries had close cooperation (Fig. 3).

### Institutions

**Fig. 4:**
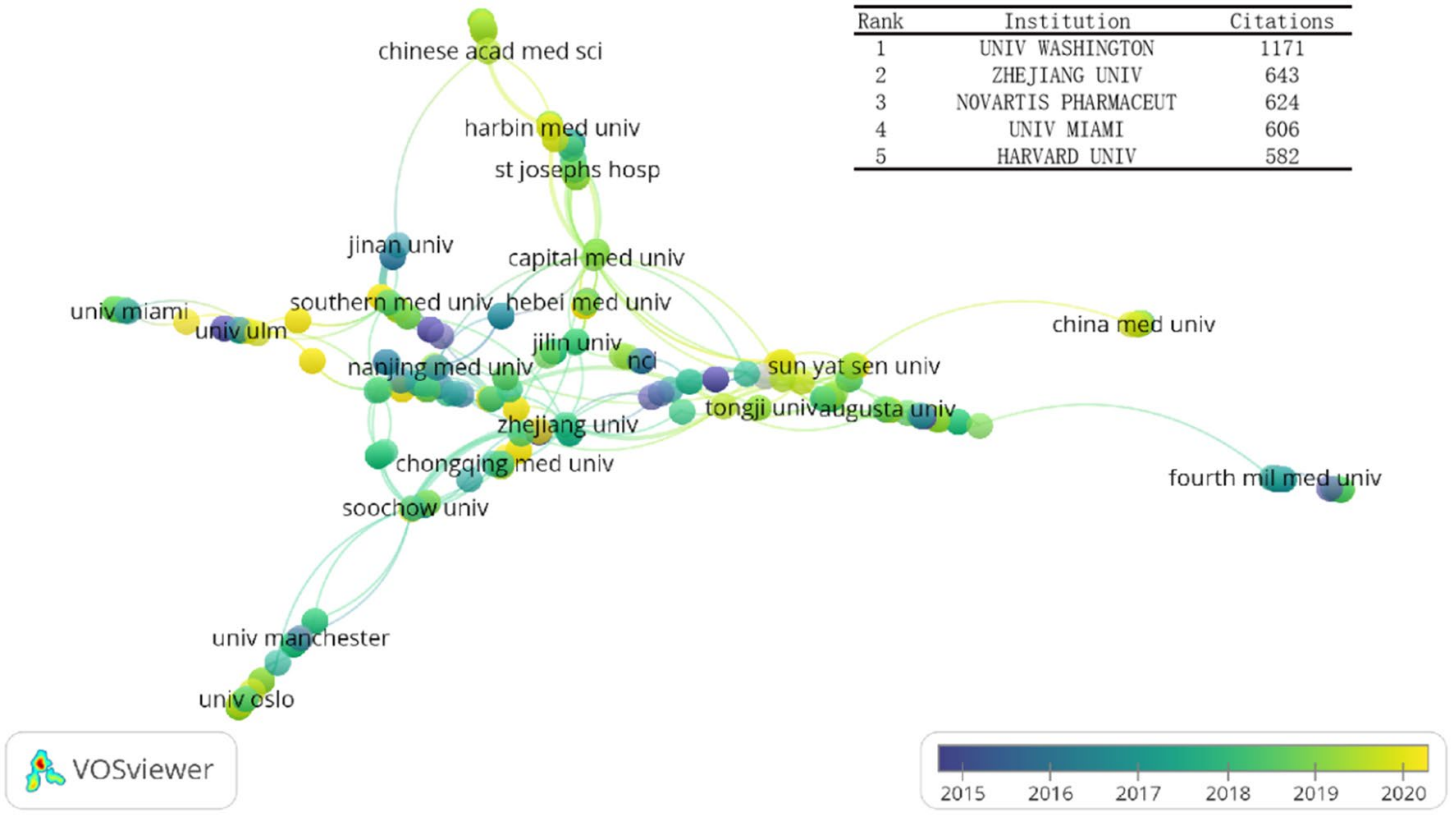
Co-authorship institutions analysis. The top 5 citations institutions and the institution co-authorship overlay visualization map. The color of each circle indicates the average publication year for the country, according to the color gradient shown in the lower right corner

We found that these publications derived from 551 institutions, among which UNIV WASHINGTON (1,171 citations) had the highest number of citations, followed by ZHEJIANG UNIV (643 citations), NOVARTIS PHARMACEUT (624 citations), UNIV MIAMI (606 citations), and HARVARD UNIV (582 citations) (Fig. 4). Furthermore, we found that more institutions have been actively involved in this field in recent years (Fig. 4; yellow denotes the recency of each institution contributing to this field).

### Key-word

**Fig. 5:**
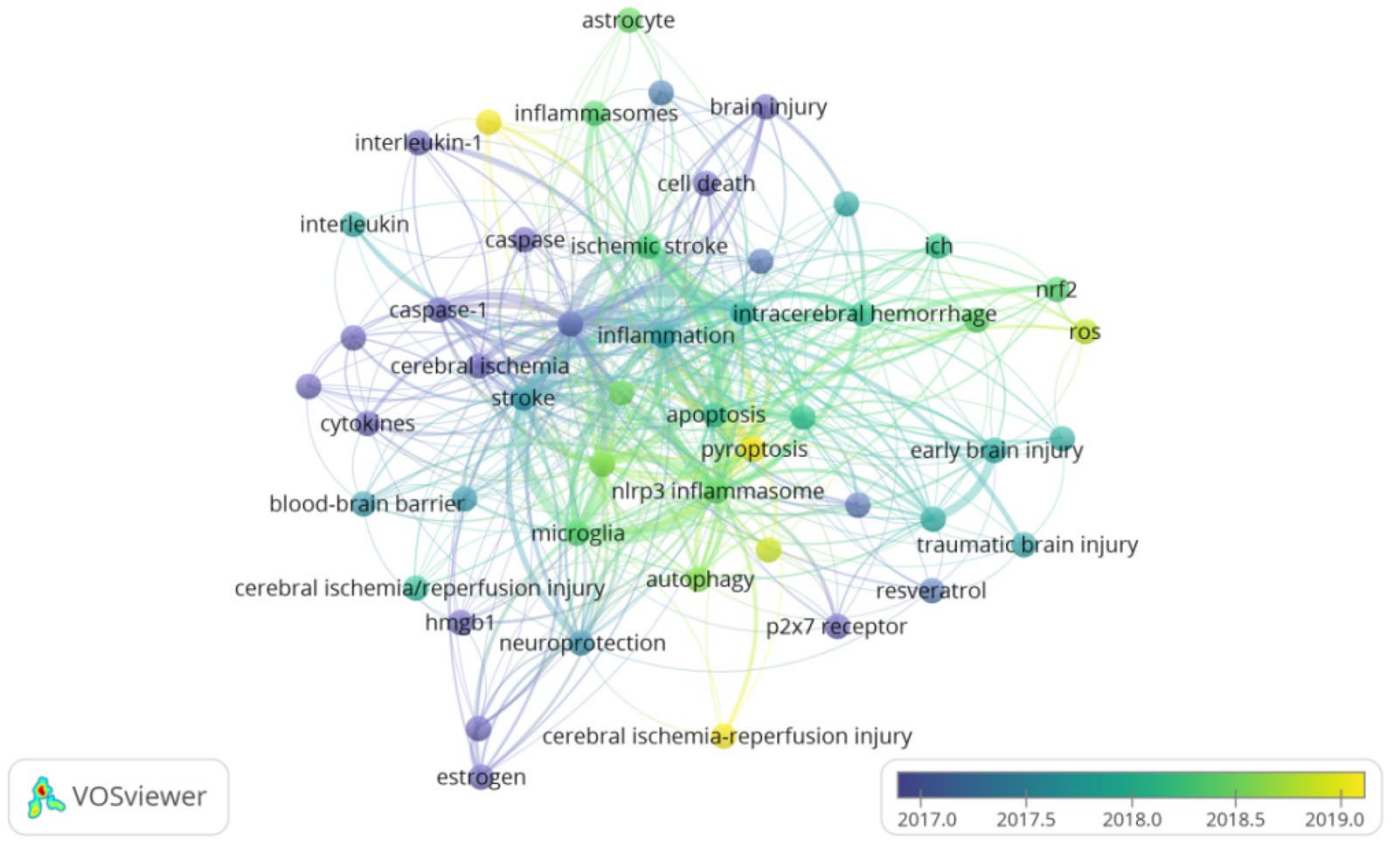
Author keyword co-occurrence analysis. Author keywords are labeled. The color of each circle indicates the average year when the keyword appeared in articles, according to the color gradient color in the lower right corner. The distance between any two circles is indicative of their co-occurrence link, and the thickness of the connecting line indicates the strength of the link

Keywords are terms that reflect the concept of a paper topic and can provide a reasonable description of the research hotspot. In recent studies, some researchers have used key-word co-occurrence networks for knowledge mapping (Fig. 5). The keywords are marked in different colors in the VOSviewer keyword concurrent visualization map, which varies according to their average publication year. Keywords such as “pyroptosis” “inflammasomes” “cerebral ischemia–reperfusion” “autophagy” “ROS” are yellow-green, indicating these fields have become popular in recent years.

## Discussion

VOSviewer is a method to manage and visualize knowledge structures [5] Here, we conducted a novel bibliometric analysis via VOSviewer regarding studies that have investigated the role of inflammasomes in stroke over the past decade to provide a comprehensive view of global research trends. Our bibliometric analysis revealed the increase in the number of articles and changes in contributions of journals, authors, countries and institutions in this field.

The number of articles in a field can reflect the productivity and development of the subject over the years. Our present results showed that the output of publications on the role of inflammasomes in stroke has maintained steady growth from 2008 to 2020. This overall growth trend in the number of related publications indicated that an increasing number of scientists were investigating the role of inflammasomes in stroke. However, a total of more than 400 articles achieved by restricting keywords, are a relatively small volume. In addition, like any other bibliometric analysis, the results have been limited by database used (WOSCC): 30% of the corpus articles identified via PubMed were excluded [8]. Despite this limitation, we believe our findings, to some extent, may contribute to the theoretical study. Furthermore, targeting inflammasomes in treating stroke has potential clinical value [28].

Citations of an article reflect the degree of its dissemination and influence, and sometimes may also reflect its quality. According to the citation analysis (Table 1), there currently is a lack of high-impact articles in this field, in which the most cited article has only 1200 citations and even 6 articles have merely been cited less than 300 times among the top 10. Alternatively, there are many information sources that can not be fully utilized, which is not expected. Therefore, it is necessary to do more work in the integration of knowledge, as the field of “inflammasomes in stroke” continues to develop. Researchers have focused on the role of inflammasomes in stroke for approximately 10 years. During this period, Bergsbaken et al. published a study in NATURE REVIEWS MICROBIOLOGY, and it was the most frequently cited article in our analysis. The authors elucidated a mechanism of action between inflammasomes and pyroptosis. The second most frequently cited article was published in AMERICAN HEART JOURNAL in 2011. The authors found that interleukins were critical mediators of the systemic inflammatory response. Activation of the NLRP3 inflammasome results in enhanced secretion of IL-1β, suggesting that therapeutic interventions targeting the assembly and activity of inflammasomes may have potential clinical benefits [25].

Popular core journals can provide a reliable reference for researchers when searching for documents. Among the journals in which “inflammasomes in stroke” related articles were published, the J NEUROINFLAMM has published the most articles, while STROKE ranks first by citations (Table 2). In terms of publications and citations, the most influential journal was STROKE, which although is the rank of eight in publication, runs first in citation. Moreover, it’s expected to provide researchers with more high-quality articles on “inflammasomes in stroke” related articles. Notably, with the help of the journals rank, researchers can quickly find the suitable journals for their own articles.

We analyzed publications from 37 countries and 551 institutions with the help of a co-authored visual map via VOSviewer. According to the map display (Fig. 3), the Unites States, China, Germany, Australia, and Singapore represented the countries that contributed most to investigating the role of inflammasomes in stroke. Among institutions, UNIV WASHINGTON contributed the most according to institutional citations (Fig. 4). These countries have invested a lot of money, manpower, and material resources in scientific research. Therefore, it is not surprising that they have become the world leaders in this field. Take China as an example: chronic diseases such as stroke, ischemic heart disease and lung cancer have now become the main causes of premature death in the Chinese population. Therefore, China has formulated a series of policies (such as Healthy China 2020 and 2030) and has continuously expanded the health care system to deal with chronic diseases such as stroke. [36] To a certain extent, government policies have greatly supported medical scientific research. Actually, it was found that the number of neuroscience publications in each country were directly proportional to their total per capita health expenditure. [35] At the same time, with the improvement of academics and research funding in recent years, developing countries (e.g., Iran, India) have also begun contributing to this field, and they have tended to exhibited close cooperation with the international community, especially with USA. Meanwhile, our analysis shows China and the United States had the closest cooperation, because there are close academic exchanges between researchers in both countries. Overseas researchers continue to collaborate within the framework of international networks after returning to their home countries.

To identify the authors who contributed the most, we ranked them based on their total numbers of citations. Based on the data extracted from WOSCC, we found that Keane’s citations ranked first, while Fann’s co-citations ranked first. These two authors, who have been recognized by the most researchers in this field, have made outstanding contributions (Table 3). Remarkably, citation is not the only indicator of the academic level, contribution, or influence of researchers, and due to the time effect, recent authors are at a disadvantage in terms of citations regardless of their content and quality. H-index is another measure of scientific influence, but it is reflected the influence of researchers in all areas, not just the in a specific field (e.g. DIETRICH WD in Table 3). Therefore, the ranking of H-index in Table 3 is inconsistent with the ranking of citation. It is necessary to conduct a regular citation analysis of inflammasomes in stroke to update the most authoritative experts in the field. Moreover, co-authorship maps may help researchers learn existing partnerships and confirm potential collaborators.

**Fig. 6:**
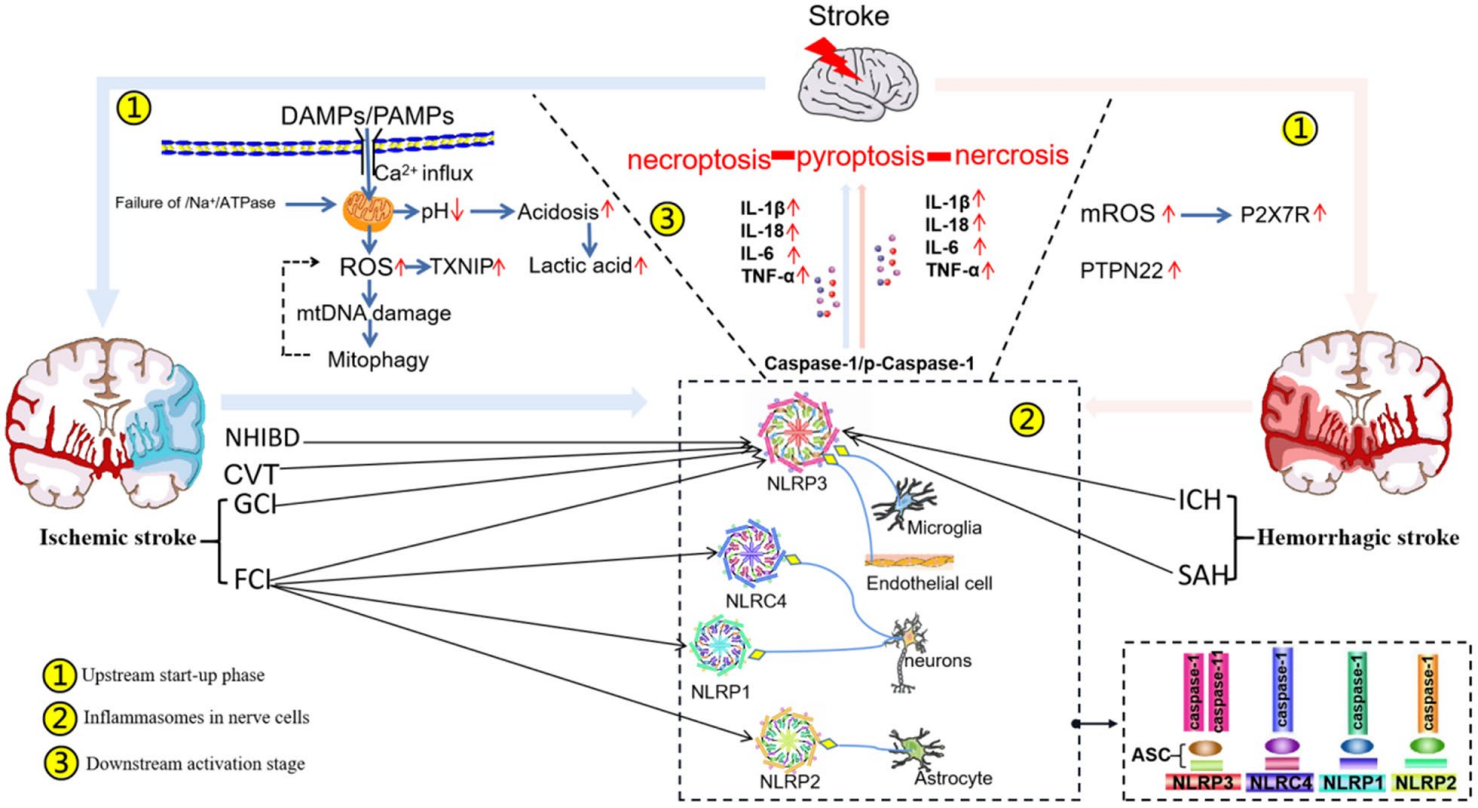
Summary of main literature. **A** Ischemic stroke (including CVT, GCI, FCI and NHIBD) and hemorrhagic stroke (including SAH and ICH) are major types of stroke. The full name is marked in the "abbreviations" section. **B** DAMPs and PAMPs lead to dysregulation of extracellular pH, efflux of Ca^2+^, failure of K^+^/Na^+^ ATPases, mitochondrial dysfunction, and DNA damage, which further more result in inflammasomes are activated. **C** Inflammasomes in general are expressed in microglia, astrocytes, neurons, and endothelial cells. Activation of inflammasomes releases excessive inflammatory cytokines, including IL-1β, IL-18, IL-6 and TNF-α, and finally contributes to cell death

According to VOSviewer key-word co-occurrence analysis and further literature reading, more information can be obtained shown in Fig. 6. It is not the content of bibliometrics in the traditional sense to collate and summarize the details of these articles. This supplementary work is intended to facilitate the reader's access to the knowledge about stroke in a more effective way. Earlier studies focused on targeting inflammasomes to treat stroke investigated mechanisms of downstream inflammatory cytokines; more recent studies then turned to investigating astrocytes, reactive oxygen species pyroptosis, and immune regulation, as well as other upstream mechanisms. More than 10 inflammasomes have been identified to be associated with stroke. These inflammasomes are expressed in microglia, astrocytes, neurons, and endothelial cells depending on the pathophysiological conditions in stroke.[29] From 2008 to 2020, a growing body of evidence has suggested that activation of inflammasomes triggers neuroinflammation through caspase-1 that further activates various downstream events (e.g. IL-1β, IL-18, IL-6 or TNF-α) and contributes to cell death. Many reviews have described various types of inflammasomes and their mechanisms of activation in stroke. The key factors responsible for activation of inflammasomes are dysregulation of extracellular pH, efflux of Ca^2+^, failure of K^+^/Na^+^ ATPases, mitochondrial dysfunction, and DNA damage [3, 12, 20, 34] Among all known inflammasomes, the most representative is the NLRP3 inflammasome, which is activated in microglia and consists of NLRP3, apoptosis-associated speck-like protein containing a caspase recruitment domain (ASC) and procaspase-1. After stroke, NLRP3 protein is abnormally activated, after which ASC is recruited. ASC then recruits procaspase-1 for its cleavage and activation, which induces the release of proinflammatory cytokines (IL-1β, IL-18, IL-6, and TNF-α) that ultimately increases local inflammation [19] If the harmful effects of inflammatory activation are not offset, the deleterious effects of stroke will become exacerbated. In addition to the NLRP3 inflammasome, stroke is also associated with the NLRP1 inflammatory inflammasome, which is composed of NLRP1, caspase-1, ASC, and X chromosome-linked inhibitor-of-apoptosis protein (XIAP), which is an inhibitor of apoptotic signaling [1, 6, 9, 10, 33] XIAP within the NLRP1 inflammasome may inhibit the activation and processing of IL-1β and IL-18 by inhibiting caspase-1 activity. Additionally, other studies have shown that stroke is also associated with NLRP2 and NLRC4 [17, 23] In view of the accelerated progress in elucidating the mechanisms of inflammasomes, their use as therapeutic targets in stroke represents a promising future clinical application. Among all potential targets, NLRP3 is the most recognized and widely implicated regulator in ischemic stroke. However, the underlying mechanisms of inflammasomes in hemorrhagic stroke have not been fully elucidated.

Unfortunately, no anti-stroke agent directly targeting inflammasomes is currently available. This may be due to insufficient evidence of the role of inflammasomes as important mediators in stroke. In addition, most studies that have investigated the biological activities of natural compounds in inflammatory bodies have been superficial. Hence, it is necessary to further elucidate the precise mechanisms of these compounds in future studies. The pharmacological effects of these natural compounds may be realized by regulating multiple targets and signaling pathways in cerebral ischemia, as opposed to only focusing on a single target. Furthermore, the activity of NLRP3 during different stages of inflammation and the relative role of NLRP3 in neonatal and adult brain inflammation remain unclear. In addition, the relationships between different inflammatory corpuscles and the relationship between inflammation and pyropotic/apoptotic cascades should also be considered. Finally, experiments characterizing the side effects of therapies targeting inflammatory bodies should also be completed.

## Conclusion

This study provided knowledge about inflammasomes in stroke from a visualization and bibliometric perspective. The results show that the research on the “inflammasomes in stroke” has been becoming progressively more extensive at global level over the past 10 years. The growing trend of publications on this topic indicates a mounting interest about it. The work focused on NLRP3 is the most systematic study, followed by NLRP1, NLRP2, and NLRC4 among the inflammasomes associated with stroke. The mechanisms about dysregulation of extracellular pH, efflux of Ca^2+^ ions, failure of K^+^/Na^+^/ATPase, mitochondrial dysfunction and mtDNA damage are source of intense research.

In general, there has been a huge advance in the field of “inflamsomes in stroke” research in the past 10 years, and it is beneficial for researchers to understand stroke better by analyzing these development trends. There are many unresolved problems about inflammasomes in stroke. As abovementioned, bibliometric analysis can provide researchers with valuable insights, and enable them to get meaningful reference based on objective data.

## Data Availability

The datasets used during this study are available from the corresponding author upon reasonable request.

## Abbreviations

NHIBD: Neonatal hypoxicischemic brain damage
CVT: Cerebral venous thrombosis
GCI: Global cerebral Ischemia
FCI: Focal ischemia
ICH: Intracerebral haemorrhage
SAH: Subarachnoid haemorrhage
NLRP3: Pyrin domain–containing protein 3
NLRP1: Pyrin domain–containing protein 1
NLRP2: Pyrin domain–containing protein 2
NLRC4: NLR family CARD domain-containing protein 4
ASC: Apoptosis-associated speck-like protein containing a caspase recruitment domain
PTPN22: Protein tyrosine phosphatase, nonreceptor type 22
P2X7R: P2X7 receptor
DAMPs: Damage-associated molecular patterns (ep.HMGB1)
PAMPs: Pathogen-associated molecular pattern (ep.LPS)
ROS: Reactive oxidative species
